# Supplementary material for: Identification of photoperiod-induced specific miRNAs in the adrenal glands of Sunite sheep (Ovis aries)
Source: Front Vet Sci. 2022 Jul 22;9:888207. doi: 10.3389/fvets.2022.888207 (PMC9354845; doi:10.3389/fvets.2022.888207)
Supplement: Supplementary file 1 [file Data_Sheet_1.ZIP › Supplementary file/miRDeep2 file.zip!/miRDeep2 file/Know_Structure/SPLP42A2/oar-mir-411b.pdf]

miRBase precursor : oar-mir-411b
Total read count : 2388
oar-miR-411b-5p read count : 2344
oar-miR-411b-3p read count : 43
remaining reads : 1

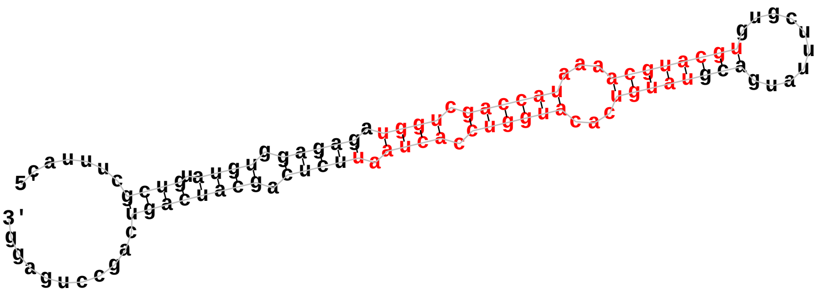

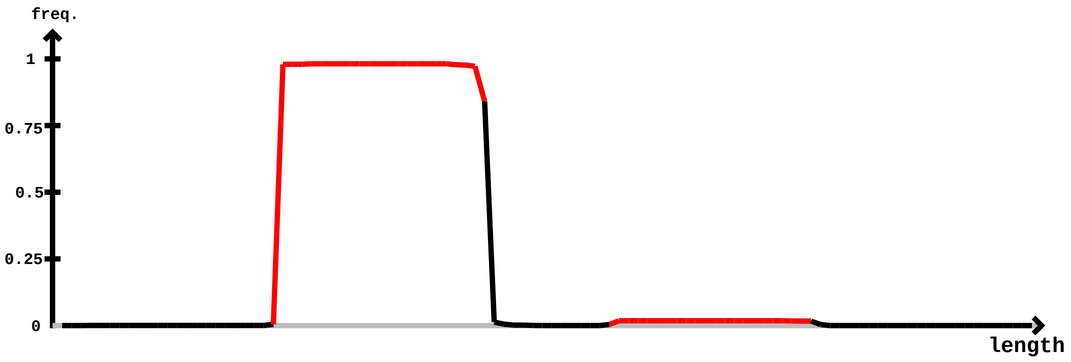


```
                                                                    oar-miR-411b-3p
                    oar-miR-411b-5p
5'- cauuucgcuguauguggagagauggucgaccauaaaacguacgugugcuuuaugacguaugucacaugguccacuaauucucagcaucagucagccugagg -3'    exp
    ......((((.((((.(((((.((((.((((((...((((((((..........))))))))...)))))).)))).))))).))))))))..........     reads    mm    sample
    ..uuucgcuguauguggagaga...............................................................................     1        0     seq
    .....................auggucgaccauaaaacguac...........................................................     1        0     seq
    .....................auggucgaccauaaaacguacg..........................................................     9        0     seq
    ......................uggucgaccauaaaacgu.............................................................     8        0     seq
    ......................uggucgaccauaaaacgua............................................................     4        0     seq
    ......................uggucgaccauaaaacguac...........................................................     9        0     seq
    ......................uggucgaccauaaaacguacg..........................................................     306      0     seq
    ......................uggucgaccauaaaacguacgu.........................................................     1975     0     seq
    ......................uggucgaccauaaaacguacgug........................................................     18       0     seq
    ......................uggucgaccauaaaacguacgugu.......................................................     9        0     seq
    .......................ggucgaccauaaaacguacgu.........................................................     1        0     seq
    .........................ucgaccauaaaacguacgugugc.....................................................     3        0     seq
    .........................ucgaccauaaaacguacgugugcu....................................................     1        0     seq
    ........................................................guaugucacaugguccacua.........................     1        0     seq
    ........................................................guaugucacaugguccacuaau.......................     7        0     seq
    ........................................................guaugucacaugguccacuaauu......................     2        0     seq
    .........................................................uaugucacaugguccacu..........................     2        0     seq
    .........................................................uaugucacaugguccacuaau.......................     24       0     seq
    .........................................................uaugucacaugguccacuaauu......................     7        0     seq
```
